# New clinical prediction model for early recognition of sepsis in adult primary care patients: a prospective diagnostic cohort study of development and external validation

**DOI:** 10.3399/BJGP.2021.0520

**Published:** 2022-04-20

**Authors:** Feike J Loots, Marleen Smits, Rogier M Hopstaken, Kevin Jenniskens, Fleur H Schroeten, Ann van den Bruel, Alma C van de Pol, Jan Jelrik Oosterheert, Hjalmar Bouma, Paul Little, Michael Moore, Sanne van Delft, Douwe Rijpsma, Joris Holkenborg, Bas CT van Bussel, Ralph Laven, Dennis CJJ Bergmans, Jacobien J Hoogerwerf, Gideon HP Latten, Eefje GPM de Bont, Paul Giesen, Annemarie den Harder, Ron Kusters, Arthur RH van Zanten, Theo JM Verheij

**Affiliations:** Julius Center for Health Sciences and Primary Care, University Medical Centre Utrecht, Utrecht University, Utrecht, the Netherlands.; Scientific Center for Quality of Healthcare, Radboud University Medical Center, Nijmegen, the Netherlands.; Bredaseweg, the Netherlands.; Julius Center for Health Sciences and Primary Care, University Medical Centre Utrecht, Utrecht University, Utrecht, the Netherlands.; Julius Center for Health Sciences and Primary Care, University Medical Centre Utrecht, Utrecht University, Utrecht, the Netherlands.; Department of Public Health and Primary Care, Katholieke Universiteit, Leuven, Belgium.; Julius Center for Health Sciences and Primary Care, University Medical Centre Utrecht, Utrecht University, Utrecht, the Netherlands.; Department of Internal Medicine and Infectious Diseases, University Medical Centre Utrecht, Utrecht University, Utrecht, the Netherlands.; Department of Clinical Pharmacy and Pharmacology and Department of Internal Medicine, University Medical Center Groningen, University of Groningen, Groningen, the Netherlands.; Faculty of Medicine, University of Southampton, Southampton, UK.; Faculty of Medicine, University of Southampton, Southampton, UK.; Unilabs Netherlands, Enschede, the Netherlands.; Rijnstate Hospital, Arnhem, the Netherlands; Rijnstate Hospital, Arnhem, the Netherlands.; Department of Intensive Care Medicine, Maastricht University Medical Centre; Care and Public Health Research Institute, Maastricht University, Maastricht, the Netherlands.; Beek, the Netherlands.; Department of Intensive Care Medicine, Maastricht University Medical Centre; School of Nutrition and Translational Research in Metabolism, Maastricht University, Maastricht, the Netherlands.; Department of Internal Medicine and Radboud Centre for Infectious Diseases, Radboud University Medical Centre, Nijmegen the Netherlands.; Emergency Department, Zuyderland Medical Centre, Heerlen; Department of Family Medicine, Care and Public Health Research Institute, Maastricht University, Maastricht, the Netherlands.; Department of Family Medicine, Care and Public Health Research Institute, Maastricht University, Maastricht, the Netherlands.; Scientific Center for Quality of Healthcare, Radboud University Medical Center, Nijmegen, the Netherlands.; Jeroen Bosch Hospital, Den Bosch, the Netherlands.; Clinical Chemistry and Haematology, Jeroen Bosch Hospital, Den Bosch; Technology and Services Research, Technical Medical Centre, University of Twente, Enschede, the Netherlands.; Gelderse Vallei Hospital, Department of Intensive Care, Ede; Division of Human Nutrition and Health, Wageningen University and Research, Wageningen, the Netherlands.; Julius Center for Health Sciences and Primary Care, University Medical Centre Utrecht, Utrecht University, Utrecht, the Netherlands.

**Keywords:** after-hours care, clinical decision rule, diagnosis, general practice, sepsis, vital signs

## Abstract

**Background:**

Recognising patients who need immediate hospital treatment for sepsis while simultaneously limiting unnecessary referrals is challenging for GPs.

**Aim:**

To develop and validate a sepsis prediction model for adult patients in primary care.

**Design and setting:**

This was a prospective cohort study in four out-of-hours primary care services in the Netherlands, conducted between June 2018 and March 2020.

**Method:**

Adult patients who were acutely ill and received home visits were included. A total of nine clinical variables were selected as candidate predictors, next to the biomarkers C-reactive protein, procalcitonin, and lactate. The primary endpoint was sepsis within 72 hours of inclusion, as established by an expert panel. Multivariable logistic regression with backwards selection was used to design an optimal model with continuous clinical variables. The added value of the biomarkers was evaluated. Subsequently, a simple model using single cut-off points of continuous variables was developed and externally validated in two emergency department populations.

**Results:**

A total of 357 patients were included with a median age of 80 years (interquartile range 71–86), of which 151 (42%) were diagnosed with sepsis. A model based on a simple count of one point for each of six variables (aged >65 years; temperature >38°C; systolic blood pressure ≤110 mmHg; heart rate >110/min; saturation ≤95%; and altered mental status) had good discrimination and calibration (C-statistic of 0.80 [95% confidence interval = 0.75 to 0.84]; Brier score 0.175). Biomarkers did not improve the performance of the model and were therefore not included. The model was robust during external validation.

**Conclusion:**

Based on this study’s GP out-of-hours population, a simple model can accurately predict sepsis in acutely ill adult patients using readily available clinical parameters.

## INTRODUCTION

Early recognition of sepsis is the critical factor influencing patient outcome.^1^^–^^4^ Protocols for the early identification of sepsis to trigger the administration of intravenous antibiotics successfully decreased sepsis-related mortality in emergency departments (EDs).^5^^,^^6^ In patients with community-acquired sepsis, GPs are often the first responding healthcare providers assessing patients.^7^^,^^8^ GPs’ recognition of sepsis and decision to refer a patient to the hospital is essential for adequate treatment. At the same time GPs have an essential role in preventing unnecessary referrals as hospital admission in itself can have a negative impact, especially in patients who are older or frail.

**Table:** How this fits in

Early recognition and treatment of sepsis are essential to improve patient outcomes. Scoring systems such as the systemic inflammatory response syndrome (SIRS), quick Sequential Organ Failure Assessment (qSOFA), and National Early Warning Score (NEWS) are used in the hospital setting for suspected sepsis but are not validated in the primary care setting. This study presents a newly developed simple score-based model that may help to predict sepsis in adult primary care patients. Biomarkers (lactate, C-reactive protein, and procalcitonin) and respiratory rate were not incorporated in this model as the added value was not clinically relevant. Before widely advocating the new model, effects on referrals and patient outcomes should be prospectively evaluated.

Currently GPs’ decisions to refer patients with severe infections to the hospital are based on an intuitive interpretation of signs, symptoms, and general impression of a patient.^9^^,^^10^ For primary care, up until now, there has been no diagnostic model available to support decisions to diagnose and manage sepsis. Clinical scores used in hospitals, like the quick Sequential Organ Failure Assessment (qSOFA),^11^ systemic inflammatory response syndrome (SIRS),^12^ or National Early Warning Score (NEWS)^13^ are not validated in primary care.

This study aimed to develop and validate a first diagnostic clinical model for the early recognition of sepsis in adults presenting in primary care. Ideally, patients with sepsis are identified early in the course of the disease and therefore the model will be designed to predict sepsis to be present within 72 hours. Immediate hospital referral is expected to improve outcome in these patients. This study investigated clinical signs, symptoms, and biomarkers potentially available at the bedside.

## METHOD

### Setting

Patients were enrolled between June 2018 and March 2020 at four participating out-ofhours primary care services in the central and south of the Netherlands (Ede, Den Bosch, Uden, and Oss). The combined area covers roughly 800 000 inhabitants in a mixed urban, suburban, and rural area. In the Netherlands, out-of-hours primary care is organised in large-scale primary care services serving between 50 000 and 400 000 inhabitants.^14^ Telephone triage is used to decide who needs to come to the clinic and who is visited at home. Only patients who received home visits were included in the study as these patients are usually more severely ill than other primary care populations. All participants (or legally authorised representatives of incapacitated patients) gave written informed consent for the study. The protocol for this study has been previously published^15^ and can be consulted for further details.

### Patients

Acutely ill adult (aged ≥18 years) patients with fever, confusion, general deterioration, or otherwise suspected severe infection were eligible for inclusion. Patients were excluded if any of the following criteria were present:

- non-infectious diagnosis suspected as the cause of the acute complaints, for example, myocardial infarction or stroke;
- hospitalisation within 7 days before the home visit;
- a condition present requiring secondary care assessment regardless of the severity of infection, for example, neutropenic fever; and
- terminal illness or other reason not to be referred to the hospital, despite the presence of a life-threatening condition.

### Candidate predictors

Based on other prediction models, sepsis guidelines, and triage protocols,^11^^–^^13^^,^^16^^,^^17^ nine clinical parameters were selected as candidate predictors. These included: age; tympanic temperature; systolic blood pressure; peripheral oxygen saturation; heart rate; respiratory rate; mental status (normal or altered); rapid progression of illness (yes/no); and rigors (yes/no). Furthermore, three biomarkers were selected: lactate; C-reactive protein (CRP); and procalcitonin (PCT).

### Procedures

The GP assessed eligibility for inclusion at the home visit. Drivers who accompanied the GPs during the home visit were equipped with portable monitoring devices (Philips Intellivue MP2 or X2) to measure blood pressure, peripheral oxygen saturation, heart rate, and respiratory rate. All vital signs and other clinical candidate predictors were registered in a case report form on site. The GP also rated the perceived likelihood of sepsis on a scale from 0–10. Either the GP or an on-call laboratory assistant obtained venous blood samples directly after inclusion. Lactate was measured by point-of-care testing (StatStrip Xpress lactate, Nova Biomedical), as lactate cannot be measured reliably from stored blood samples.^18^ The venous blood samples were stored at −70°C for later measurements of CRP and PCT. All patients received care as usual.

### Outcome definitions and assessment

Three expert panels were created, each consisting of one GP, one emergency physician, and one intensivist (or acute care internist). These expert panels established the primary outcome: ‘sepsis within 72 hours of inclusion’, using all relevant information from medical records, per Sepsis-3 definition.^19^ The operational definition of sepsis is the presence of infection and a SOFA score^20^ at least two above the baseline. Cases were divided among the three panels, with 10% of all cases being evaluated by all three panels for inter-rater and inter-panel reliability. If panel members could not reach a consensus on the presence or absence of sepsis, the case was discussed in a face-to-face meeting until consensus was reached.

Secondary outcomes assessed by the expert panel included whether the infection was the cause of acute complaints (yes/no) and the need for hospital treatment (on a scale from 0–10). Furthermore, the presence or absence of an ‘adverse outcome’, defined as an intensive care unit (ICU) admission within 72 hours or death within 30 days of inclusion, was determined.

### Statistical analysis

Baseline characteristics of the study population were described using the mean and standard deviation for continuous variables with a normal distribution; and the median and interquartile range (IQR) for variables with a skewed distribution. Inter-rater and inter-panel reliability were assessed using Cohen’s kappa for the primary outcome of sepsis.

Multiple imputations using multivariate imputation by chained equations (MICE) procedure^21^^,^^22^ were used to account for missing data. The regression coefficients and performance measures of the imputed datasets were pooled using Rubin’s rules^23^ and the total covariance matrix, respectively.

First, a multivariable logistic regression model was developed using all clinical parameters. Subsequently, lactate, CRP, and PCT were added to this clinical model. The linearity of the relationship between continuous variables and the log odds of sepsis was assessed. For non-linear relationships, a restricted cubic spline was used. Backward selection with *P*<0.157 as selection criterion (based on the Akaike information criterion)^24^^,^^25^ was used to remove any non-informative clinical parameters and biomarkers. Subsequently, performance measures of the model with and without biomarkers were compared using predictors as continuous variables (hereafter referred to as the ‘continuous model’). Variables included in this model were then dichotomised, creating a simplified model based on a simple count of the number of predictors. All cut-off points of vital signs used in NEWS were considered for the simplified model, and the model with the highest C-statistic (equal to the area under the receiver operating curve) was chosen as the final model.

Combined with previously described methods for imputing missing data and variable selection,^26^ optimism was calculated to adjust for C-statistics of the continuous models using tenfold cross-validation. The calibration slope was used as a shrinkage factor for model regression coefficients and subsequently re-estimating the intercept.

Discrimination was evaluated using the C-statistic. Calibration was assessed by visual inspection of the calibration plots and by evaluating the calibration slope and Brier score. In addition, the calibration of external datasets was also assessed using the observed-to-expected (O/E) ratio as a measure for mean calibration. Percentiles of bootstrapped samples were used to calculate 95% confidence intervals (CIs) for performance measures. Performance measures of the continuous and simplified model were compared to each other, as well as to the performance of existing scoring systems, that is, SIRS, qSOFA, and NEWS, and to the likelihood of sepsis (on a scale from 0–10) according to the GP on site.

R (version 4.0.5) package was used for the analyses.

### Sensitivity analyses

Model performance for secondary outcomes was assessed to evaluate potential incorporation bias resulting from the use of the SOFA score (by the expert panel) as part of the sepsis definition, as well as for a more conservative calculation of the SOFA score (fewer SOFA points for decreased oxygen saturation and altered mental status).

### External validation

Datasets from patients with suspected infections assessed in two Dutch EDs were used to test the external validity of both the continuous and simplified model. The C-statistic discrimination was assessed, and the continuous and simplified models were compared with NEWS. The calibration was assessed using calibration plots, as well as mean calibration and calibration slope. A more detailed description can be found in Supplementary Appendix S1.

## RESULTS

### Study population and outcome

**Figure 1.**
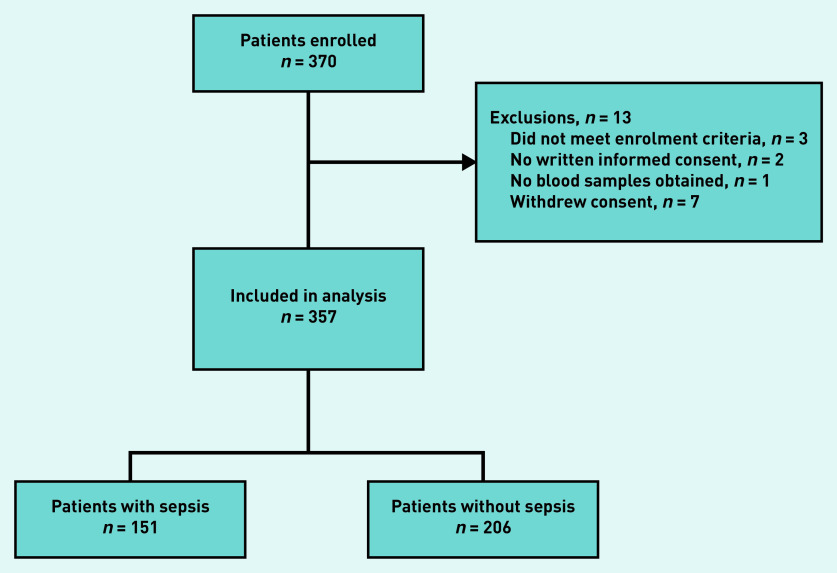
*Patient flow chart.*

In total, 357 patients were included for analysis (Figure 1). The median age was 80 years (IQR 71–86) and 61% were male (Table 1). The GPs referred 199 patients (56%) to the ED directly after inclusion, of which 188 (94%) were subsequently admitted to hospital. Of the 158 patients not referred immediately, 22 (14%) were admitted to hospital within the first 72 hours after inclusion (data not shown). Of the 357 patients included in the analysis, 12 (3.4%) were admitted to the ICU within 72 hours after inclusion, and overall 30-day mortality was 5.6%. The proportion of missing values was low for all candidate predictors, with the highest being 3.6% for PCT. A total of 151 patients (42%) had sepsis, according to the expert panel. Cohen’s kappa, indicating the inter-rater reliability between members within the same panel, ranged between 0.57 and 0.76 (mean 0.68), and Cohen’s kappa for inter-panel reliability ranged between 0.69 and 0.95 (mean 0.79) (see Supplementary Table S1). Table 1 shows a summary of the characteristics of patients with and without sepsis.

**Table 1.** Patient characteristics by sepsis diagnosis, *N* = 357

**Characteristic**	**Patients with sepsis (*n*= 151)**	**Patients without sepsis (*n*= 206)**
**Demographics**		
Age, years, median (IQR)	80 (74–85)	79 (68–86)
Sex, *n* (%)		
Male	93 (62)	123 (60)
Female	58 (38)	83 (40)

**Comorbidities, *n* (%)**		
Diabetes	55 (36)	49 (24)
COPD	22 (15)	40 (19)
Cardiac disease	63 (42)	59 (29)
Cerebrovascular accident	33 (22)	39 (19)
Malignancy	19 (13)	30 (15)
Chronic kidney disease	43 (28)	49 (24)
Dementia	25 (17)	18 (8.7)
Immunosuppressive use	6 (4.0)	7 (3.4)

**Final diagnosis, *n* (%)**		
Respiratory tract infection	61 (40)	74 (36)
Urinary tract infection	45 (30)	47 (23)
Abdominal infection	12 (7.9)	7 (3.4)
Skin/soft tissue infection	11 (7.3)	17 (8.3)
Infection with unknown source	11 (7.3)	25 (12)
Other source of infection	11 (7.3)	8 (3.9)
Non-infectious diagnosis	—	28 (14)

**Candidate predictors**		
Tympanic temperature, °C, mean (SD)	39.0 (0.7)	38.5 (1.0)
Systolic blood pressure, mmHg, mean (SD)^a^	135 (25)	139 (24)
Heart rate, beats/min, mean (SD)	100 (20)	96 (20)
Respiratory rate, breaths/min, mean (SD)^a^	26 (6)	23 (7)
Peripheral oxygen saturation, %, median (IQR)^b^	93 (90–95)	95 (93–97)
Altered mental status, *n* (%)	81 (54)	46 (22)
Rigors, *n* (%)	100 (66)	123 (60)
Rapid illness progression, yes, *n* (%)	127 (84)	144 (70)
Lactate, mmol/L, median (IQR)^a^	1.6 (1.1–2.1)	1.3 (0.9–1.7)
C-reactive protein, mg/L, median (IQR)^c^	85 (34–145)	57 (20–114)
Procalcitonin, ng/mL, median (IQR)^d^	0.25 (0.09–1.20)	0.08 (0.03–0.22)
Time to blood collection, minutes, median (IQR)	50 (26–65)	45 (15–65)

**Secondary outcomes, *n* (%)**		
Hospital admission	134 (89)	76 (37)
Length of stay, days, median (IQR)	5.2 (3.1–8.3)	4.5 (2.5–6.5)
ICU admission within 72 hours	11 (7.3)	1 (0.5)
30-day mortality	13 (8.6)	8 (3.9)

a *Missing,* n *= 1.*

b *Missing,* n *= 2.*

c *Missing,* n *= 6.*

d *Missing,* n *= 13. COPD = chronic obstructive pulmonary disease. ICU = intensive care unit. IQR = interquartile range. SD = standard deviation.*

### Prediction model development

Of the nine clinical candidate predictors, six were included in the continuous model after backward selection: aged >65 years; temperature >38°C; systolic blood pressure ≤110 mmHg; respiratory rate; peripheral oxygen saturation ≤95%; and mental status. Age was included as a restricted cubic spline with three knots. After correction for optimism, the continuous model without biomarkers had a C-statistic of 0.80 (95% CI = 0.75 to 0.84), a calibration slope of 0.86, and a Brier score of 0.181 (See Supplementary Table S2 for regression coefficients). The addition of the three biomarkers to this model resulted in lactate and PCT remaining after backward selection. However, the optimism corrected C-statistic of 0.80 was identical to the model without biomarkers. Therefore, no biomarkers were included in the final continuous model. Analyses of individual biomarkers are shown in Supplementary Figure S1.

A simplified model was created through the dichotomisation of variables included in the continuous model (Box 1). Models without respiratory rate were also evaluated, as the respiratory rate is less feasible for GPs to perform. Heart rate showed collinearity with respiratory rate, and model performance did not decrease after substitution. Consequently, heart rate was used instead of respiratory rate in the final simplified model.

**Box 1.** Simplified model of six variables, resulting in a score ranging between 0–6 points

Aged>65 years	1 point
Tympanic temperature >38 °C	1 point
Systolic blood pressure ≤110 mmHg	1 point
Heart rate>110 beats/minute	1 point
Peripheral oxygen saturation ≤95%	1 point
Altered mental status	1 point

**Figure 2.**
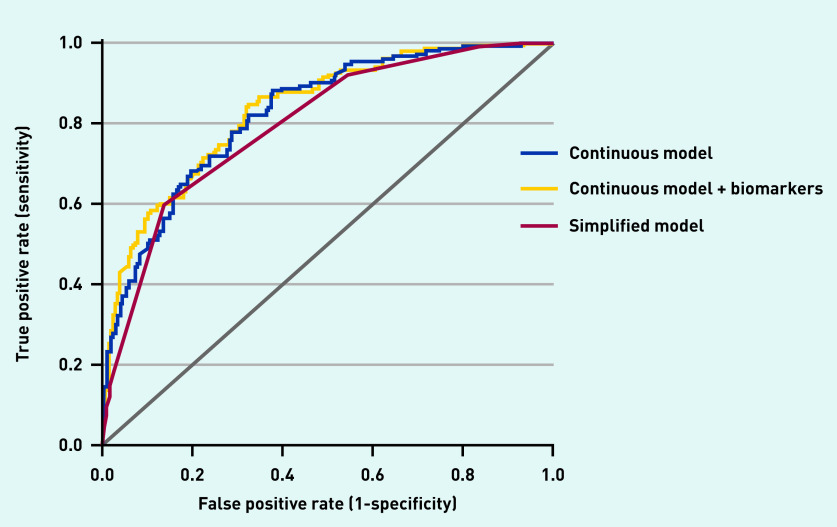
*Receiver operating curves of the continuous model, continuous model + biomarkers (lactate and procalcitonin), and simplified model for sepsis outcome.*

**Figure 3.**
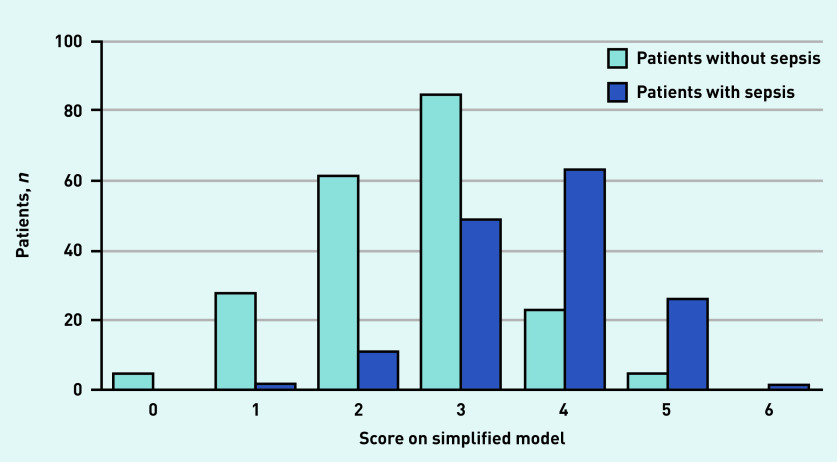
*Number of patients with and without sepsis for all scores on the simplified model.*

**Figure 4.**
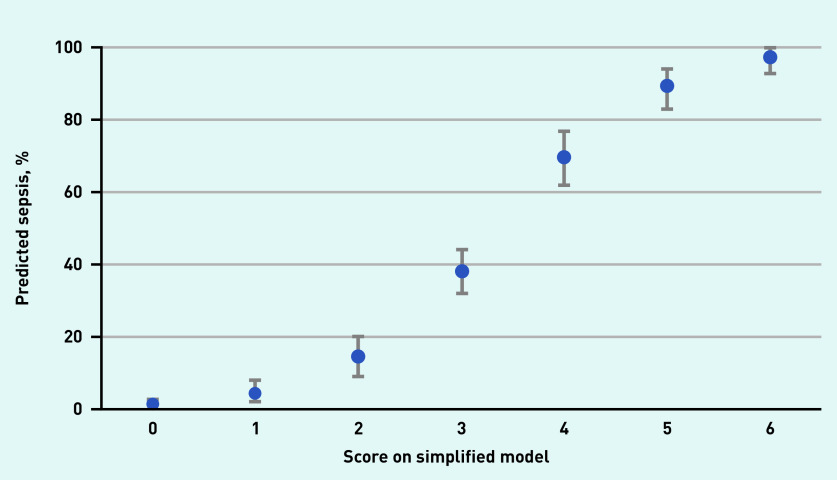
*Predicted rate of sepsis with 95% confidence intervals for all scores on the simplified model.*

Discrimination of the simplified model (C-statistic of 0.80, 95% CI = 0.76 to 0.83) was nearly identical to the continuous model (Figure 2). Diagnostic accuracy measures for the simplified model at different cut-off scores are presented in Table 2, distribution of the patients with and without sepsis are shown in Figure 3, and predicted rate of sepsis in Figure 4. The calibration of the simplified model was also similar to the continuous model (see Supplementary Figure S2). The use of multiple cut-off points for individual variables in the model, or grouping score categories, did not significantly improve performance.

**Table 2.** Diagnostic accuracy measures with 95% confidence intervals of the simplified prediction model for predicting sepsis at different score thresholds in development data, *N* = 357

**Cut-off point, (*n*)**	**Sensitivity (95% CI)**	**Specificity (95% CI)**	**LR+ (95% CI)**	**LR–(95% CI)**	**PPV (95% CI)**	**NPV (95% CI)**
≥1 (352)	100 (98 to 100)	2.4 (0.8 to 5.6)	1.02 (1.00 to 1.05)	0.00	43 (38 to 48)	100
≥2 (324)	99 (96 to 100)	16 (11 to 21)	1.18 (1.11 to 1.25)	0.04 (0.01 to 0.31)	46 (41 to 52)	97 (84 to 100)
≥3 (251)	92 (87 to 96)	46 (39 to 53)	1.69 (1.48 to 1.93)	0.17 (0.10 to 0.31)	55 (49 to 62)	89 (81 to 94)
≥4 (118)	60 (51 to 68)	86 (81 to 91)	4.39 (3.03 to 6.34)	0.47 (0.38 to 0.57)	76 (68 to 84)	74 (68 to 80)
≥5 (32)	18 (12 to 25)	98 (94 to 99)	7.37 (2.90 to 18.7)	0.84 (0.78 to 0.91)	84 (67 to 95)	62 (56 to 67)

*LR+ = positive likelihood ratio. LR–= negative likelihood ratio. PPV = positive predictive value. NPV = negative predictive value.*

### Comparison with existing models

**Figure 5.**
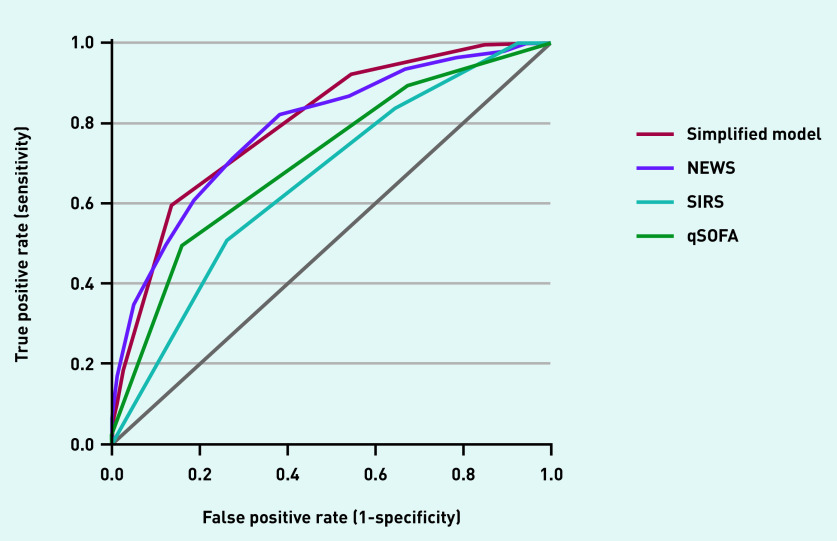
*Receiver operating curves of the simplified model, NEWS, qSOFA, and SIRS for the outcome sepsis. qSOFA = quick Sequential Organ Failure Assessment. NEWS = National Early Warning Score. SIRS = systemic inflammatory response syndrome.*

Performance of the continuous and simplified models was compared to SIRS, qSOFA, and NEWS (Table 3). NEWS showed similar performance compared to the simplified model with a C-statistic of 0.79 (95% CI = 0.75 to 0.83). SIRS and qSOFA had lower C-statistics of 0.66 (95% CI = 0.61 to 0.70) and 0.71 (95% CI = 0.66 to 0.75), respectively (Figure 5). The perceived probability of sepsis within 72 hours by the GP on site resulted in a C-statistic of 0.73 (95% CI = 0.67 to 0.78; data not shown). Brier scores for continuous and simplified models were lower, that is, better than SIRS, qSOFA, and NEWS.

**Table 3.** Optimism corrected performance measures in development data of the multivariable model consisting of clinical parameters as continuous variables (continuous model), with the addition of lactate and procalcitonin (continuous model + biomarkers), simplified model, SIRS, qSOFA, and NEWS, *N* = 357

**Prediction model**	**C statistic (95% CI)**	**Calibration slope**	**Brier score**
Continuous model	0.80 (0.75 to 0.84)	0.86	0.181
Continuous model + biomarkers	0.80 (0.74 to 0.84)	0.83	0.176
Simplified model	0.80 (0.76 to 0.83)	1.00	0.175
NEWS	0.79 (0.75 to 0.83)	1.01	0.182
qSOFA	0.71 (0.66 to 0.75)	1.02	0.207
SIRS	0.66 (0.61 to 0.70)	1.03	0.224

*qSOFA = quick Sepsis-related Organ Failure Assessment. NEWS = National Early Warning Score. SIRS = systemic inflammatory response syndrome (based on criteria: temperature* <*36°C or* >*38°C, respiratory rate* >*20 breaths/min, and heart rate* >*90 beats/min*).

### Sensitivity analyses

The prediction of secondary endpoints (including an alternative sepsis definition using more restrictive calculation of the SOFA score) resulted in comparable performance results for the continuous model, simplified model, and NEWS for all analyses. C-statistics ranged between 0.7 and 0.8 for all outcomes, except for prediction of ‘adverse outcome’ (ICU admission <72 hours or 30-day mortality), where a C-statistic of 0.58 (95% CI = 0.51 to 0.66) was found for the continuous model, compared to 0.62 (95% CI = 0.53 to 0.69) for both the simplified model and NEWS (see Supplementary Table S3).

### External validation

The first validation dataset (Dataset 1) was from a teaching hospital in the south of the Netherlands and previously published by Latten *et al*.^7^ The population consisted of 440 patients with a median age of 71 years, of whom 163 (37%) were diagnosed with sepsis (severe sepsis or septic shock according to the Sepsis-2 definitions).^27^ A C-statistic of 0.80 (95% CI = 0.77 to 0.83) was found for the simplified model and 0.84 (95% CI = 0.80 to 0.87) for the continuous model. Calibration showed an O/E ratio of 1.4 for the simplified model and 1.5 for the continuous model.

The second dataset (Dataset 2) from an academic medical centre in the north of the Netherlands consisted of 1340 patients with a median age of 65 years, of whom 342 (26%) were diagnosed with sepsis (Sepsis-3 criteria). In this dataset, the C-statistic was 0.70 (95% CI = 0.67 to 0.72) for the simplified model and 0.70 (95% CI = 0.67 to 0.73) for the continuous model. The O/E ratio was 1.4 for the simplified model and 1.7 for the continuous model (see Supplementary Tables S4 and S5, and Supplementary Figures S3–S5 for complete external validation results).

## DISCUSSION

### Summary

In this observational cohort study, a new and easy-to-use prediction model was developed for the early recognition of sepsis in primary care. Biomarkers provided no significant improvement in prediction performance when added to the model. The respiratory rate could be replaced with the more accessible and more reliable measure, heart rate, without decreasing the prediction performance of the simplified model. The performance of the simplified model was significantly better than SIRS and qSOFA. The outcomes of the present study’s simplified model were comparable to NEWS.

The validity of the simplified model was confirmed in the external validation, though some differences were found in discrimination and calibration compared to development data.

Three different aspects may have contributed to these discrepancies. First, the outcome ‘sepsis’ was defined differently in the external datasets. The SIRS-based sepsis definition may have introduced incorporation bias in the first external dataset (Dataset 1), resulting in better NEWS predictions. Second, the variable ‘altered mental status’ was registered differently. Any empirical change in mental status was sufficient in the presented study cohort, while a decrease in the Glasgow coma score was used in the validation cohorts. This score is probably less sensitive to subtle changes in mental status. Finally, admission of intravenous fluids and supplemental oxygen by ambulance personnel to patients with sepsis have likely occurred. Consequently, vital signs may have normalised once patients arrived at the ED and were included in the study.^28^

A simple score-based model can accurately predict sepsis in adult primary care patients with suspected severe infections at home. Biomarkers do not improve the model’s predictive performance. The score does not replace clinical judgement, and further research will have to demonstrate how GPs can best use the score to improve the management of patients with possible sepsis.

### Strengths and limitations

To the authors’ knowledge, this study is the first to include patients in their home situation, where the decision to refer the patient had yet to be made. This is a major strength as the potential impact on patient care is larger in these patients than in patients already in, or in transit to, the hospital. Another strength of the study is the prospective design, specifically tailored to developing a clinical prediction rule. As only very few data on the candidate predictors were missing, the study was sufficiently powered according to prevailing sample size calculation methods.^25^^,^^29^^,^^30^ Furthermore, the newly developed models were internally and externally validated and compared to existing scoring systems.

Several limitations of this study should be taken into account. First, using an expert panel as a reference standard for sepsis may have resulted in biased results. Verification bias may have occurred as patients referred to the hospital received more diagnostic tests than non-referred patients. Second, as some candidate predictors were also part of the SOFA score, this may have resulted in incorporation bias. Therefore, sensitivity analyses were performed, using a stricter calculation of the SOFA score and alternative outcomes, that is, adverse outcomes and need for hospital treatment according to the expert panel. These analyses did not suggest significant bias. Furthermore, not all eligible patients had been included in the study. However, the most common reasons not to include eligible patients were not based on patient factors but rather on having too busy a shift, which is unlikely to have resulted in selection bias. Finally, the external validations were performed in patients assessed in the accident and emergency department due to suspected infection. Ideally, validation of the model would have been performed in a primary care population in whom the decision to refer a patient to the hospital was not yet made. These data were not available to the authors. However, the fact that the model also performed well in other domains underscores robustness.

### Comparison with existing literature

Other clinical prediction rules have been proposed for either patients with sepsis or those who are critically ill in the prehospital setting. These were mostly derived from retrospective data retrieved from patients transported by ambulance and used SIRS-based sepsis definitions.^31^ Only one prospective cohort study using the Sepsis-3 outcome definition was found in the prehospital setting, which included 551 patients with suspected infection in the ambulance.^32^ This study showed blood pressure ≤100 mmHg, temperature >38.5 °C, lactate >4 mmol/L, gastrointestinal symptoms, and altered mental status to be most predictive of sepsis. These findings mainly align with the results of the present study and support the decision not to include respiratory rate in the simplified model. In the present data, only three patients showed lactate >4 mmol/L, which might explain why lactate was not found to be a useful predictor in the primary care setting. Two studies were found in which vital signs were measured in acutely ill adult patients in a primary care setting.^32^^,^^33^ However, both studies only included patients who were referred to a hospital or acute care clinic, and both did not report sepsis as an outcome measure.

The simplified prediction model developed in the current study was comparable to NEWS. NEWS was initially developed for the early detection of clinical deterioration of adult patients admitted to the hospital.^34^ Recent studies in the ED setting showed NEWS superior to SIRS and qSOFA in predicting sepsis,^35^^,^^36^ which was confirmed in the present study for the primary care setting. An implementation study of NEWS in the prehospital setting in England showed promising results,^37^ but NEWS was only performed in 30% and 63% of cases by GP-support teams and ambulance personnel, respectively.^38^

### Implications for research and practice

Though the difference between empirical clinical assessment by the GP and performance of the present model was modest, it can help support clinicians during the busy daily routine, reduce variation in the quality of primary care, and improve collaboration between primary and secondary care for this potentially life-threatening condition. The model is not intended to overrule the GP’s overall judgement but rather to inform the GP on the probability of the sepsis outcome. The GP can subsequently use this information to decide whether or not to refer the patient to hospital. The presented simplified model is easy to use in daily practice. Compared to the NEWS score, the presented model does not include respiratory rate and does not have a complex scoring matrix. The results do not mean that respiratory rate should not be measured in severely ill patients, and the minority of the GPs who are currently using NEWS are using a valid and useful model, as the present results showed. The simplified model presented here showed similar diagnostic properties and could be easier to implement in the primary care setting. After the decision to refer a patient owing to suspected sepsis, ambulance personnel can score the NEWS depending on local protocols. Before widely advocating the new model, effects on referrals and patient outcomes should also be prospectively evaluated in a pragmatic trial in primary care.

## Data

De-identified patient data that underlie the presented results in this article will be made available to researchers with a methodological sound proposal. A data dictionary of all variables will be provided. Data will be available from 3 months after publication until 5 years after publication. Requests should be made to the corresponding author.
